# Clinical efficacy of nivolumab is associated with tertiary lymphoid structures in surgically resected primary tumors of recurrent gastric cancer

**DOI:** 10.1371/journal.pone.0262455

**Published:** 2022-01-07

**Authors:** Takuya Mori, Hiroaki Tanaka, Sota Deguchi, Yoshihito Yamakoshi, Yuichiro Miki, Mami Yoshii, Tatsuro Tamura, Takahiro Toyokawa, Shigeru Lee, Kazuya Muguruma, Masaichi Ohira

**Affiliations:** Department of Gastroenterological Surgery, Osaka City University Graduate School of Medicine, Osaka, Japan; Hamad Medical Corporation, QATAR

## Abstract

Nivolumab, an immune checkpoint blocker, has been approved for advanced gastric cancer (GC), but predictive factors of nivolumab’s efficacy in patients with GC, especially immune cells such as tissue-resident memory T cells or those forming tertiary lymphoid structures (TLS), remain unclear. Tissue samples were obtained from surgically resected specimens of patients with GC who were treated with nivolumab as third-line or later treatment. Immunohistochemical staining was performed to detect the presence of TLS and CD103^+^ T cells and assess the association between TLSs and response to nivolumab treatment. A total of 19 patients were analyzed. In patients with partial response (PR) to nivolumab, numerous TLS were observed, and CD103^+^ T cells were found in and around TLS. Patients with many TLS experienced immune-related adverse events more often than those with few TLS (*p* = 0.018). The prognosis of patients with TLS high was better than those with TLS low. Patients with a combination of TLS high and CD103 high tended to have a better prognosis than other groups. Our results suggested that TLS status might be a predictor of nivolumab effectiveness.

## 1. Introduction

Advanced gastric cancer (GC) has a poor prognosis, despite the development of various chemotherapies [1–3]. Immunotherapy approaches, especially immune checkpoint blockade of programmed death 1 (PD-1), has recently emerged as a promising strategy in the treatment of several cancers [4–6].

Nivolumab, a human IgG4 monoclonal antibody against PD-1, demonstrated survival benefit in patients with advanced GC in a Phase 3 trial (ATTRACTION-2) [7] and has been applied in the treatment of advanced GC.

As immunotherapy has well-recognized clinical efficacy, research to identify biomarkers that predict efficacy has also progressed. In GC, tumor-infiltrating lymphocytes (TILs) are known to be associated with favorable prognosis [8]. It has been reported that Epstein-Barr positive GC, patients with high microsatellite instability, and high tumor mutational burden exhibit increases in local TILs and clinical response to pembrolizumab [9–11]. However, it is difficult to directly quantify TILs accurately; thus, the development of a simpler evaluation method is desirable.

Tertiary lymphoid structures (TLS), aggregates of various immune cells around B cells, are structurally and functionally similar to secondary lymph organs, and function as antigen-preserving cells and activate T cells [12, 13]. TLS are associated with antitumor immune responses and good prognosis in several cancers [14–16]. We also previously reported that TLS in GC can be easily and objectively quantified and correlated with prognosis [17]. Cabrita et al. [18] recently reported that TLS improve the efficacy of immunotherapy against melanoma and therefore could be a biomarker for nivolumab. In addition, Workel et al. [19] reported that CD103^+^ T cells, which are tissue-resident memory T cells that have attracted attentions as an immunotherapy target [20–22], produced CXCL13 which is essential for the formation of TLS [12]. Moreover, we previously showed that CD103^+^ T cells are considered to be a subpopulation of CD8^+^ TILs [23]. We hypothesized that the density of TLS around the primary tumor may help predict the clinical response to nivolumab therapy in patients with advanced GC in replace of CD8^+^ TILs.

The aim of the present study is to assess the association between the presence of TLS and the response to nivolumab in patients with recurrent GC.

## 2. Materials and methods

### 2.1. Patients and samples

This study included patients with advanced GC who had been treated with nivolumab as third- or later line therapy at Osaka City University Hospital between 2017 and 2020. The standard nivolumab dose of 3 mg/kg was administrated intravenously every 2 weeks until disease progression or unacceptable toxicity. We obtained 19 tumor tissue samples from surgically resected specimens and evaluated them retrospectively. Patients with R1 surgery (peritoneal metastasis (P1) or intraoperative peritoneal lavage cytology positive (P0CY1)) were included if tumor lesions were resected macroscopically. Patients who underwent chemotherapy before surgery were excluded. Pathological tumor-node-metastasis (TNM) staging was determined histologically based on the 7^th^ edition of the Union for International Cancer Control TNM classification, and the histologic type was determined based on the 14^th^ Edition of the Japanese Classification of Gastric Cancer. Differentiated (well differentiated, moderately differentiated, or papillary adenocarcinoma) and undifferentiated (poorly differentiated, mucinous adenocarcinoma, or signet-ring cell carcinoma) types were defined according to the predominant histologic type in the tumor. Response of the target region to nivolumab was classified according to the Response Evaluation Criteria in Solid Tumors guidelines. This study was performed according to the Declaration of Helsinki and approved by the Osaka City University Ethics Committee. Informed consent was obtained from all patients.

### 2.2. Immunohistochemistry

TLS is observed as aggregates of various immune cells, including B cells, T cells, high endothelial venules, and follicular dendritic cells, and the centers of TLS are located in B cells that formed clusters. We therefore defined the cluster of CD20^+^ B cells as TLS in this study, as we previously reported [17]. All immunohistochemistry analyses were carried out on 4-μm-thick sections from paraffin-embedded tumor blocks. Nonspecific binding was blocked using nonspecific staining blocking reagent (Dako, Agilent Technologies, Inc.). The sections were then reacted with mouse anti-CD20 (clone: L26; prediluted; Dako, Agilent Technologies, Inc.) or rabbit monoclonal anti-CD103 (clone: EPR4166 (2); 1/1000; Abcam) antibody at 4°C overnight. Sections were incubated with secondary antibody for 10 min at room temperature. After washing in phosphate-buffered saline, the sections were visualized using 3–3’-diamino-benzidine for 5 min and counterstained with hematoxylin.

### 2.3. Evaluation of immunohistochemical staining

Tumor sections stained with anti-CD20 antibody were scanned at ×20 magnification. Three fields with the greatest area of intratumoral and peritumoral CD20^+^ cells were randomly selected, and the percentage area (%) of each field with CD20^+^ cells was calculated using Image J software (NIH, Bethesda, MD, USA). Tumor sections stained with anti-CD103 antibody were scanned at ×200 magnification. The five most representative high-power fields were randomly selected and the average number of CD103^+^ T cells in the five fields was calculated. All microscopic images were imported from the digital photo filing system DP-73 (Olympus, Tokyo, Japan), and immunohistochemical analyses were performed by two observers who blinded to clinical outcome. In case of disagreement, the slides were reviewed and reanalyzed until a consensus was reached between two observers. Patients were divided into two groups according to the median range: TLS high and TLS low, CD103 high and CD103 low.

### 2.4. Statistical analysis

Statistical analysis was performed using JMP 14 software (SAS Institute, Cary, NC, USA). Fisher’s exact test was used to assess the associations between the expression of CD20 and clinicopathological features. Overall survival (OS) and progression-free survival (PFS) curves were calculated according to the Kaplan-Meier method, and significant differences in survival were determined using the log-rank test. The day of nivolumab start was used as the starting point for the measurement of OS. PFS was defined as the time between the day of each chemotherapy start and disease progression. For all other experiments, data were compared using the two-tailed Student’s *t*-test. For all analyses, *p*-values < 0.05 were considered statistically significant.

## 3. Results

### 3.1. Patients

A total of 19 patients (7 female and 12 male) were analyzed, and the median age was 69 (60–76) years. There were 8 patients of stage IV (2 P1, 6 P0CY1), and these cases were R1 surgery although tumor lesions were resected macroscopically. The median time from surgery to disease recurrence and to initiation of nivolumab treatment was 9.2 (6.5–18.2) and 18.7 (11.0–35.1) months, respectively. The median nivolumab administration time was 4.9 (1.9–9.3) months. Four patients experienced immune-related adverse events (irAEs) (1 pneumonia, 1 gastrointestinal perforation, 1 hypothyroidism, 1 adrenal gland dysfunction). 2 patients experienced irAEs within 3 months, and 2 patients experienced irAEs after 8 months. Partial response (PR) was observed in 3 patients, stable disease (SD) in 5 patients, and progressive disease (PD) in 11 patients; and the response rate was 15.8% and disease control rate (DCR) 42.1%. Among the 4 patients with irAEs, PR was observed in 2 patients, SD in 1 patient, and PD in 1 patient. The OS period was 7.0 (3.9–15.2) months, and the PFS period was 4.9 (2.1–9.3) months.

**Fig 1:**
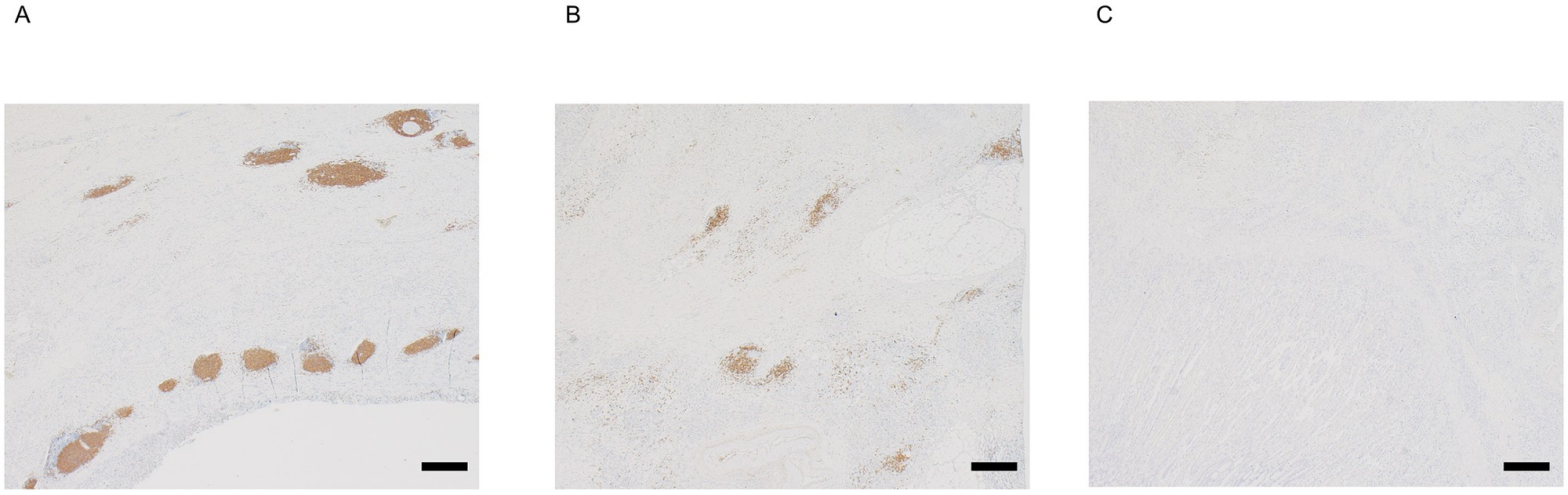
Representative immunohistochemical images of TLS. (A) Predominant staining pattern: the percent area of TLS was 2.9%. (B) Median range: the percent area of TLS was 1.24%. (C) Predominant nonstaining pattern: the percent area of TLS was 0%. Scale bars: 500 μm.

Representative images of TLS are shown Fig 1. The median percentage area of TLS in the 19 patients was 1.24% (0.31%-2.14%) and the values for patients with PR and irAEs are shown in S1 Fig.

### 3.2. Clinicopathological features between patients with TLS high and low

The association between TLS and the clinicopathological features is summarized in Table 1. There were no associations between the density of TLS and age, gender, performance status, histological type, pathological progression, or the number of CD103^+^ T cells. Moreover, no significant difference in time from initiation of 1st-line chemotherapy to nivolumab between patients of TLS high and low (high vs low, 18.0 vs 12.9 months, *p* = 0.624). For nivolumab treatment, there was no difference between TLS high and low and nivolumab treatment duration. However, patients with TLS high experienced irAEs more often than those with TLS low (*p* = 0.033). All patients exhibiting PR were high TLS. DCR in patients with TLS high and low was 66.7% and 20%, respectively, and patients with TLS high tended to have a better response to nivolumab treatment than those with TLS low (*p* = 0.063).

**Table 1:** Correlations between clinicopathological factors and Tertiary Lymphoid Structures (TLS).

		n or median	TLS		
			low	high	*p*
Age	<75 years	12	6	6	1.0
	≥75 years	7	4	3	
Gender	Female	7	2	5	0.350
	Male	12	8	4	
Performance status	0	5	3	2	0.794
	1	9	4	5	
	2	5	3	2	
Histology	Differentiated	6	3	3	1.0
	Undifferentiated	13	7	6	
pT status	2–3	6	4	3	1.0
	4	13	6	6	
pN status	0	3	2	1	1.0
	1–3	16	8	8	
pStage	I-III	11	5	6	0.650
	IV	8	5	3	
The number of CD103^+^ T cells		22.2 (10.2–42.2)	18.7 (7.4–42.6)	25.2 (18.3–39.9)	0.191
Target for treatment					
Liver metastasis		3	3	0	
Lymph node metastasis		5	2	3	0.261
Peritoneum metastasis		11	5	6	
Time from surgery to disease recurrence (month)		9.2 (6.5–18.2)	12.3 (6.6–32.6)	9.0 (6.0–13.9)	0.327
Time from initiation of 1st-line chemotherapy to nivolumab (month)		14.7 (7.0–28.2)	12.9 (6.4–29.2)	18.0 (9.2–27.2)	0.624
Nivolumab administration time (month)		4.9 (1.9–9.3)	3.9 (2.1–7.0)	4.9 (1.6–9.7)	0.683
irAE	-	15	10	5	0.033
	+	4	0	4	
Response to treatment	PR	3	0	3	0.062
	SD	5	2	3	
	PD	11	8	3	

TLS; Tertiary lymphoid structure, irAE; Immune-related Adverse Event, PR; Partial Response, SD; Stable Disease, PD; Progressive Disease

Data are expressed as median range (interquartile range) or n.

### 3.3. Survival analysis

**Fig 2:**
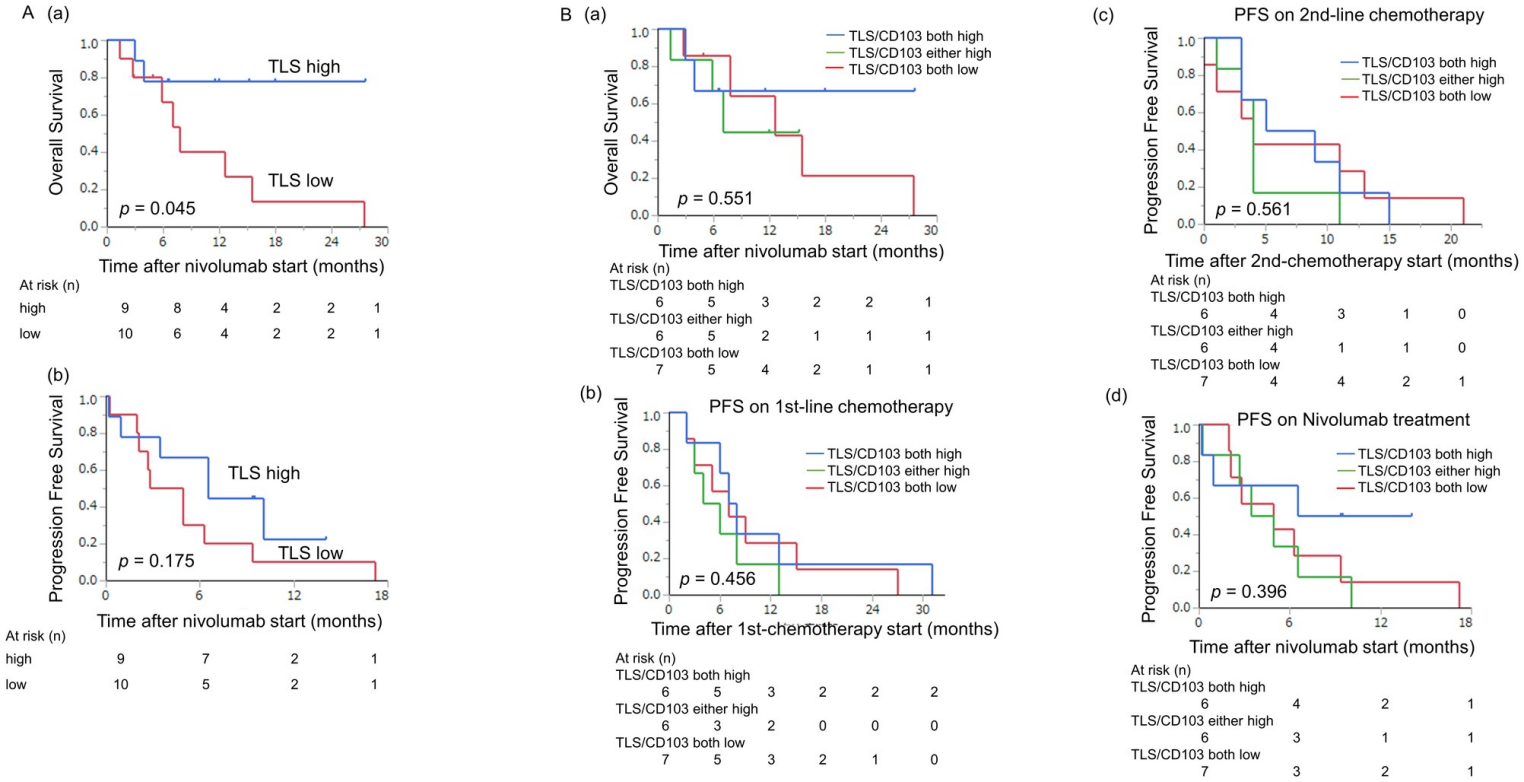
Prognostic impact of TLS in GC. (A) Kaplan-Meier plots using the log-rank test for OS and PFS according to the percent area of TLS. Patients with TLS high had a better prognosis than those with TLS low (a: OS; *p* = 0.045). Patients with TLS high tended to have a better prognosis (b: PFS; *p* = 0.1752). (B) Kaplan-Meier plots using the log-rank test for OS according to the combination of TLS and CD103^+^ T cells. (a) Patients with TLS high and CD103 high tended to have a better prognosis than other groups. (b-d) There was not a statistically significant difference in PFS on 1st- and 2nd-line chemotherapy and nivolumab therapy. When nivolumab therapy was used as the starting time of PFS, individuals with high TLS and high CD103 tended to have longer PFS.

Patients with TLS high had improved OS (Fig 2A, a; *p* = 0.045). Although no significant difference in PFS was observed, median PFS was 6 months in the TLS low group versus 9 months in the TLS high group (Fig 2A, b). When considering the combination of TLS and CD103^+^ T cells, patients with TLS high and CD103 high had a tendency to be longer survival compared with the other groups (Fig 2B, a). For each treatment period, there was not a statistically significant difference in PFS on 1st- and 2nd-line chemotherapy and nivolumab therapy. On the other hand, when nivolumab therapy was used as the starting time of PFS, individuals with high TLS and high CD103 tended to have longer PFS (Fig 2B, b-d).

### 3.4. Representative case presentation

**Fig 3:**
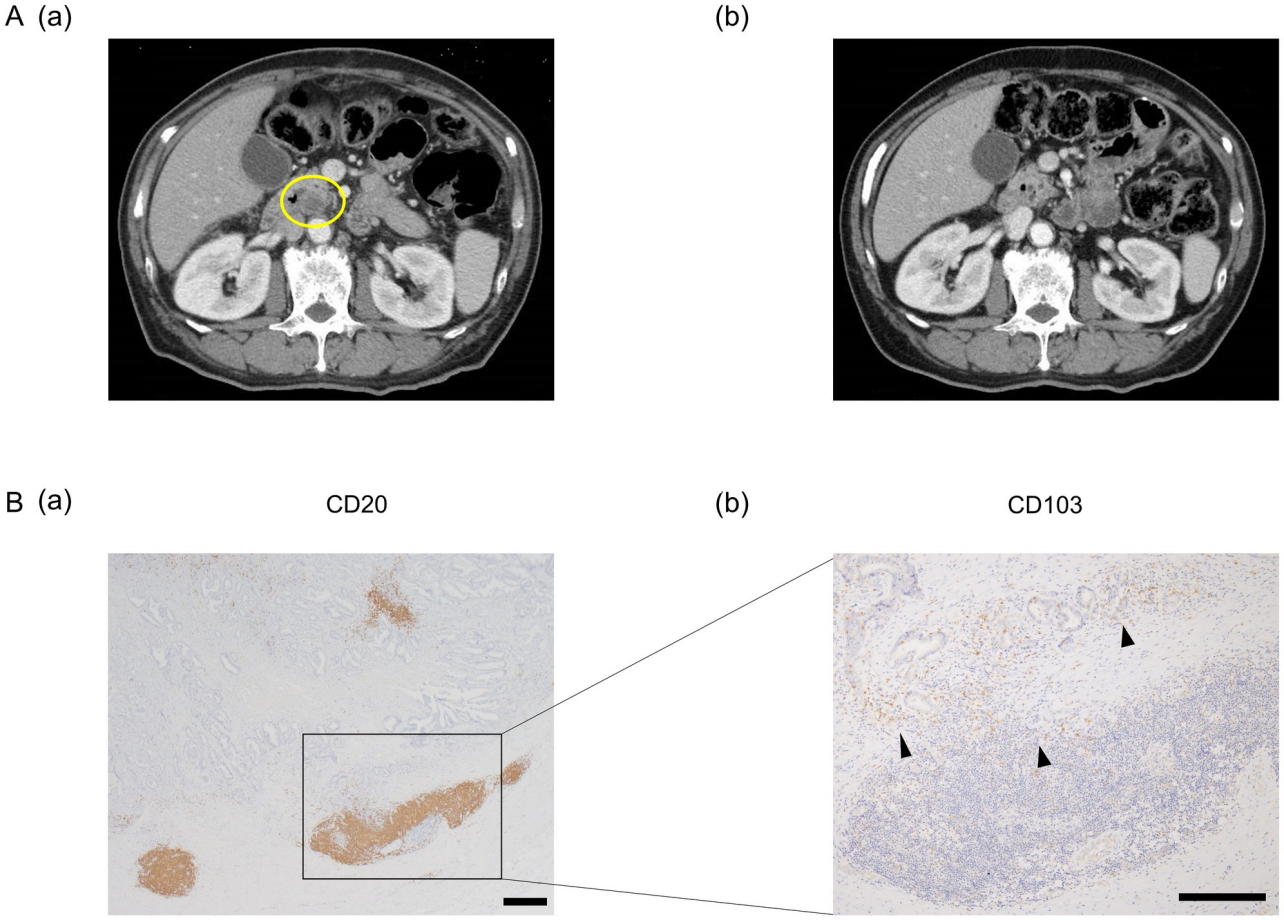
Representative CT-scan images and immunohistochemistry results using anti-CD20 and anti-CD103 antibodies in a PR patient. (A, a) Para-aortic lymph node metastasis recurrence was found (yellow circle). (b) Six months later, the metastatic lymph node decreased. (B, a) Aggregates of CD20^+^ cells were observed in the tumor tissue, which were considered TLS. Scale bar: 500 μm. (b) CD103^+^ T cells were found around TLS (black arrowhead). Scale bar: 500 μm.

We show a representative example of patients who had a PR. The patient with pT3N1M0f stage IIIA received nivolumab as third-line treatment for para-aortic lymph node metastasis recurrence. Six months later, the lymph node metastasis had decreased (Fig 3A), and we evaluated as PR. Finally, the patient was treated with nivolumab for 14 months. Immunohistochemistry analysis of tumor tissue from this patient revealed large aggregates of CD20^+^ cells, which were referred to as TLS, were observed in the tumor tissue (Fig 3B, a). The percentage of area of TLS was 2.36%. Furthermore, numerous CD103^+^ T cells were located around the TLS in this patient (Fig 3B, b).

## 4. Discussion

In this study, we showed that patients with TLS high experienced more irAEs and had a better response to nivolumab treatment and better prognosis than those with TLS low in terms of prognosis of recurrent GC treated with nivolumab in third and subsequent lines.

In this study, we found that patients with recurrent GC treated with nivolumab, in whom TLS were found predominantly around the periphery of the primary tumor, exhibited longer survival time after nivolumab treatment, even if they had SD or PD. From this result, it can be inferred that the immune response is more likely to occur or be enhanced in the patient’s body. An important component of the local immune response is the TIL population. The immune cycle proposed by Chen et al. [24] recognizes the importance of lymphoid tissues in the process of naive T cells being presented with antigens and become cytotoxic T lymphocytes (CTLs); i.e., CD8^+^ TILs. We previously showed that TLS in GC may function as antigen-presenting cells and have the ability to induce CTLs [25].

On the other hand, various studies had shown that CD8^+^ T cells expressing CD103 are associated with a good prognosis in some cancers including GC [22, 26–29]. CD103 is a marker of tissue-resident memory T cells which are considered potent effector cells. We also demonstrated that approximately 70% of CD8^+^ TILs in GC are resident memory T cells [23]. Furthermore, in murine model, it was reported that CD103^+^ T cells produce CXCL13, which is essential for the formation of TLS [12, 30]. Therefore, we hypothesized that TLS could be used for evaluation in GC instead of TILs.

Some recent studies showed that TLS enhance the response to immunotherapy in patients with melanoma or lung cancer and therefore might serve as a biomarker of anti PD-1 therapy [18, 31]. CD103^+^ T cells themselves express PD-1 [20], and previous studies showed that CD103^+^ T cells might be a suitable target of anti PD-1 therapy [22, 32]. As shown in the case presentation, the patient with PR exhibited TLS high and CD103 high. Although no significant difference was found between the density of TLS and CD103^+^ T cells in this study because of small sample size, the median number of CD103^+^ T cells was 18.7 in TLS low versus 25.2 in TLS high, and patients with TLS high and CD103 high tended to predict a longer prognosis only on nivolumab treatment. In other words, we speculated that the presence of both TLS and CD103^+^ T cells is associated with a strong immune response and is actually predictive of outcome to immunotherapy.

Other indirect evidence of a strong immune response to nivolumab in patients with high TLS is in relation to irAEs. Several studies have shown that irAEs are associated with the efficacy of PD-1 blockade in patients with various cancers [33–36]. The development of irAEs is thought to be bystander effect by activated T cells. In other words, when effective cases, T cells which activated by immunotherapy attack not only tumor cells but also normal tissues, resulting in the development of irAEs [37]. We speculate that the activation of T cells is associated with TLS. Indeed, in the present study, 4 patients experienced irAEs and all patients had TLS high. Moreover, of the 4 patients, 3 patients predicted a good disease control by nivolumab therapy. Although there is a possibility that prolonged treatment with nivolumab in patients with TLS high leads to the development of irAEs, some patients developed irAEs early. Therefore, our results suggest that patients with TLS high mount a stronger immune response and a higher T cell activation than those with TLS low, resulting in more irAEs and better effectiveness of nivolumab.

This study has several limitations. First, the study was retrospective in design and had a small sample size of only 19 patients from a single institution. Furthermore, this study contains many patients who underwent surgery for radical resection. Therefore, these results may not apply to all patients who receive 3rd-line or higher nivolumab therapy, particularly to patients with de novo metastasis. Although it is clear that further confirmation in large scale cases is needed, this is the first report suggesting that local TLS in gastric cancer may be associated with the prognosis of patients treated with immune checkpoint molecules. Second, the tumor immune environment could have changed during nivolumab administration may change from the environment present when we evaluated TLS using surgically resected specimens as chemotherapy was introduced before nivolumab treatment for GC. Although Silina et al. [38] showed that neoadjuvant chemotherapy impairs the maturation of TLS, whether the area of TLS changed before and after chemotherapy remains unclear, because it is difficult to evaluate TLS using biopsy samples. Third, TLS were evaluated only by immunohistochemistry and thus, intratumoral heterogeneity could have affected the results. However, the ability to detect TLS by immunohistochemistry in low-power fields is an advantage. In the future, research on another evaluation method such as blood markers that reflect intratumoral TLS by analyzing cells-free DNA using liquid biopsy is desired to apply to patients with de novo metastasis.

## 5. Conclusions

TLS around the resected tumor were found to be associated with superior OS but not PFS in nivolumab treatment after recurrence in 19 patients with GC. This suggests that an anti-tumor immune response may be generated by TLS around the primary tumor. The number of local TLS may therefore be evaluated in the future as a predictor of nivolumab efficacy in the treatment of recurrent GC.

## Supporting information

S1 Fig Histogram of the percentage area of TLS in 19 patients. The median percentage area of TLS in the 19 patients was 1.24% (0.31%-2.14%). The percentage area of TLS in the patients with PR and irAEs is 2.39% and 2.01%, respectively.
(TIF)
