# Supplementary figures and images for: Clinical efficacy of nivolumab is associated with tertiary lymphoid structures in surgically resected primary tumors of recurrent gastric cancer
Source: PLoS One. 2022 Jan 7;17(1):e0262455. doi: 10.1371/journal.pone.0262455 (PMC8741034; doi:10.1371/journal.pone.0262455)

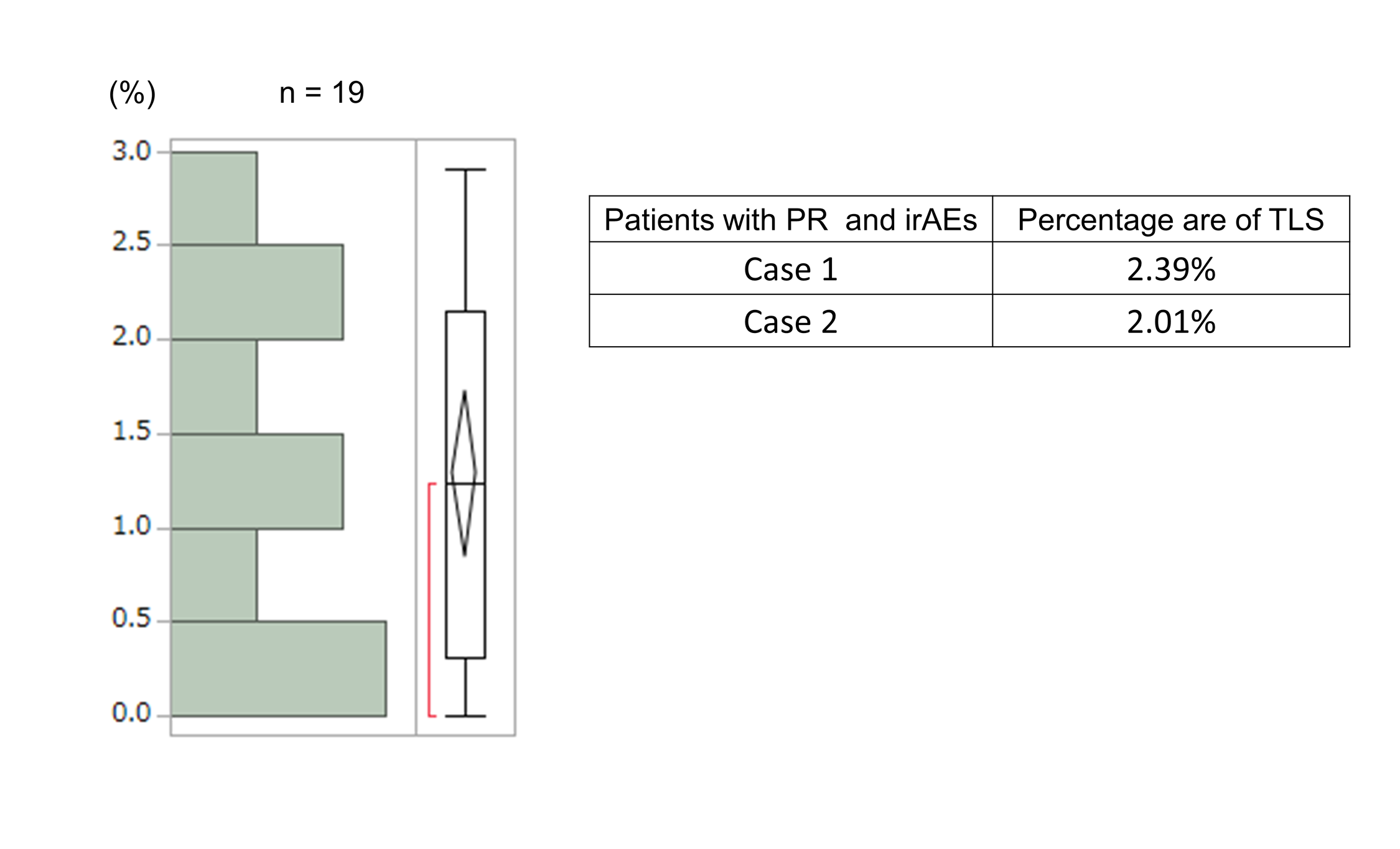

Supplement: S1 Fig — The median percentage area of TLS in the 19 patients was 1.24% (0.31%-2.14%). The percentage area of TLS in the patients with PR and irAEs is 2.39% and 2.01%, respectively. (TIF) [file pone.0262455.s001.tif]
